# Weight Status and Behavioral Problems among Very Young Children in Chile

**DOI:** 10.1371/journal.pone.0161380

**Published:** 2016-09-01

**Authors:** Rose M. C. Kagawa, Lia C. H. Fernald, Jere R. Behrman

**Affiliations:** 1 School of Public Health, University of California, Berkeley, CA, United States of America; 2 Department of Economics, University of Pennsylvania, Philadelphia, PA, United States of America; McGill University, CANADA

## Abstract

**Background/Objectives:**

Our objective was to explore the association between weight status and behavioral problems in children before school age. We examined whether the association between weight status and behavioral problems varied by age and sex.

**Subjects/Methods:**

This study used cross-sectional data from a nationally-representative sample of children and their families in Chile (N = 11,207). These children were selected using a cluster-stratified random sampling strategy. Data collection for this study took place in 2012 when the children were 1.5–6 years of age. We used multivariable analyses to examine the association between weight status and behavioral problems (assessed using the Child Behavior Checklist), while controlling for child’s sex, indigenous status, birth weight, and months breastfed; primary caregiver’s BMI and education level; and household wealth.

**Results:**

Approximately 24% of our sample was overweight or obese. Overweight or obese girls showed more behavioral problems than normal weight girls at age 6 (*β* = 0.270 SD, 95% CI = 0.047, 0.493, *P* = 0.018). Among boys age 1 to 5 years, overweight/obesity was associated with a small reduction in internalizing behaviors (*β* = -0.09 SD, 95% CI = -0.163, -0.006, *P* = 0.034).

**Conclusions:**

Our data suggest that the associations between weight status and behavioral problems vary across age and sex.

## Introduction

Worldwide, an estimated 2.1 billion children and adults (30% of the population) are overweight.[1] Among preschool-age children, the average prevalence across all countries is relatively low (6%),[2] but prevalence reaches as high as 11.7% in some higher-income countries.[3] Chile, similar to a number of other high-income countries, is experiencing elevated and rapidly increasing rates of overweight and obesity, most notably among preschool- and school-age children.[4] Recent estimates put the prevalence of overweight or obesity among preschool-age children in Chile at 8.2% and among first graders at 18.5%.[5]

Obesity during childhood is associated with the development of numerous diseases in the short and long terms, including type II diabetes and cardiovascular disease,[6,7] and some psychosocial problems, though most overweight and obese children do not develop serious mental health issues.[6–10] The association between weight status and behavioral problems varies across age and sex,[7,8] as well as by type of sample (e.g. clinical vs. nonclinical).[9,10]

Child psychopathology researchers divide child behavioral issues into externalizing and internalizing problems. Externalizing problems are marked by under-control of emotions (e.g. aggressive conduct and attention problems) while internalizing problems are marked by over-control of emotions (e.g. anxiety, depression and withdrawal).[11]

Among the externalizing problems, attention-deficit/hyperactivity disorder (ADHD) is most commonly associated with elevated weight status among children. A systematic review of the literature showed that obesity and ADHD are possible comorbidities, especially among clinical samples.[12] However, evidence about the association between weight status and other externalizing behavioral issues is mixed, and appears to vary by age. Studies among children over 8 years old and adolescents, for example, have found associations between excess weight and at least one measure of externalizing problems such as conduct disorder,[13] oppositional defiant disorder,[14] delinquent behavior,[15] or bullying.[16,17] Other research suggests that there is no association between weight status and externalizing disorders in younger children.[18–20]

In addition to a possible increase in externalizing issues, overweight and obese children may also be at higher risk of internalizing disorders such as anxiety[21–23] and depression.[22,24] Again, the association between weight and anxiety or depression among children is quite heterogeneous and some studies show no association.[20,25] Of the internalizing problems hypothesized as comorbidities of elevated weight status, however, body dissatisfaction demonstrates the most consistent association with overweight and obesity.[26–29]

Elevated weight status and emotional and behavioral problems may be linked before children enter school. For example, in a study of U.S. children at the beginning of kindergarten, overweight girls, but not boys, had higher levels of teacher-reported internalizing and externalizing problems compared to normal weight peers.[30] According to another study, weight status and externalizing behaviors were positively correlated as early as 24 months of age.[31] Very few existing studies include children pre-kindergarten, and findings from studies of preschool-age children show mixed results.[31–33]

Several mechanisms have been proposed to explain the association between child weight status and behavioral problems. Poor impulse control may lead to an overconsumption of food,[34] subsequent weight gain,[35] and a greater exhibition of externalizing behaviors, particularly ADHD.[36] Increased sedentary behavior and emotional eating have been linked to internalizing behaviors,[37] which may be a form of compensating for an inability to cope with negative emotions.[38] Child behavior may also influence how and what parents feed their children. For example a study found that mothers fed more sweet foods and drinks to infants exhibiting either or both internalizing and externalizing behaviors, perhaps in an effort to calm them.[39] Weight-based stigma is commonly hypothesized to mediate the association between weight and psychological problems,[40] and children are stigmatized for being overweight as early as age 3 years.[41] These explanations include common causes of weight and behavior, pathways that indicate weight status precedes behavioral problems, and pathways that indicate the opposite–that behavioral problems precede weight.

The temporal ordering of weight gain and the development of behavioral problems has not been well established. Psychological problems in childhood or adolescence have been shown to be predictive of the onset of overweight among children followed two years later,[42] and also predictive of adult obesity.[24,43] The opposite has also been found, however, with weight status predicting psychosocial problems later in life.[44,45] Thus, obesity seems to be a “dynamic process” in which weight status interacts with genetic, emotional, behavioral, cognitive and social factors, rather than a “stable condition.”[9]

As described in detail above, most studies of childhood obesity and psychopathology have focused on school-age children and adolescents (5 to 18 years of age).[6] Very few include children who have not yet entered kindergarten; notable exceptions include two studies using data from the National Institute of Child Health and Human Development Study of Early Child Care and Youth Development.[31,46] Similarly few studies take place outside of Western countries, with most occurring in European countries, Australia, and North America.[6]

The current study adds to the literature by focusing on an under-studied developmental stage (early childhood) in this literature and by considering a Latin American context, Chile. The objective of this study is to explore the association between weight status and behavioral problems in very young children. Specifically we examine whether sex and age modify the association between weight status and behavioral problems. Based on prior research, our hypothesis is that the associations between weight and behavioral problems become stronger across early childhood, and that this pattern is more notable among girls.

## Methods

### The Data

This study uses a cross-section of publicly available de-identified data from the Chilean *Encuesta Longitudinal de la Primera Infancia* [Chilean Longitudinal Study of Early Infancy] (ELPI), which is a nationally-representative sample of 18,310 children born in Chile between 2006 and 2011, and their families (data available from: http://www.microdatos.cl/Encuestas/PrimeraInfancia/LongitudinalPrimeraInfancia). These children were selected using a cluster-stratified, random-sampling strategy, using the municipality as the primary sampling unit. The sampling frame included all registered births occurring between January 1st, 2006 and August 31st, 2009. Municipalities were stratified by socioeconomic level and selected using systematic random sampling with probability of selection proportionate to size. Municipalities covering the country’s largest cities received a sampling probability of one to ensure inclusion. Children in municipalities were selected using systematic random sampling from the Civil Registry until the desired sample size for each municipality was reached. Data collection began in 2010 with interviewers visiting the homes of the selected children to collect data on physical, cognitive and socio-emotional development indicators; family background; and participation in early childhood development programs. Follow-up data were collected in 2012, at which time a supplementary sample of children born between the two rounds was added. Interviews were conducted with the primary caregivers of the children, who were the mothers of the children in 99% of households. The primary caregivers of the selected children provided voluntary and written consent to participate in the study. The ELPI was supported by the Chilean Ministries of Education and Labor,[47,48] and the Institutional Review Board at the University of Pennsylvania approved this research.

In our analyses, we used the second wave of the ELPI, when the children were 1.5 to 6 years of age. To participate in the behavioral assessment, children needed to be 1.5 years of age or older. The behavioral assessment was applied during a separate follow-up interview after the initial questionnaire was applied. As a result, for children that did not complete the behavioral assessment, we had their birthdate but not their assessment date. Therefore, we could not be certain of their age at the point in time when they would have taken the test had they in fact taken it and could not assess age-eligibility directly. To address this, we created a birthdate cut-off based on the ages of the children that did take the test to determine age eligibility for the children that did not take the test. Of the 17,209 age-eligible children, a total of 13,315 were assessed for psychological and behavioral problems (measures described below). Of children assessed for psychological and behavioral problems, 10,716 had complete covariate data. Missing child’s current weight (8% of total observations), birth weight (8% of total observations) or length of breastfeeding (5% of total observations) were the primary causes of this reduction in sample size.

For missing covariate data, we substituted values from the child’s baseline measurement in 2010 when available, which brought the sample size up to 11,207. To assess the potential for non-representativeness arising from the loss of observations with missing data, we compared key demographic variables of children in the sample to those excluded because of missing data.

We conducted a post-hoc power calculation to estimate the minimum detectable effect given the study’s sample size. Using an alpha level of 0.05, a sample size of 11,207 distributed unequally between exposed and unexposed groups, and taking into account the clustering of individuals within communities, this study had 80% power to detect a difference of 0.019 standard deviations.

### Measures

#### Weight status

Our main exposure variable was weight status. After receiving formal training on proper measurement of anthropometrics, interviewers weighed and measured the children during visits to the homes. Children under 2 years of age were weighed in the arms of their caretaker and the caretaker’s weight was subsequently subtracted. Children over 2 years old and adults were weighed individually. The interviewers asked caretakers to remove their shoes and other bulky items (e.g. jackets) before weighing. Children were weighed without diapers, wearing a maximum of two light items of clothing. A SECA brand digital scale was used for all weight measurements. To measure length, children under 2 were measured lying down. A parent assisted the researcher in keeping the child still and straight, and the interviewer used a ruler or book and masking tape to mark the child’s length against a measuring tape attached to a flat surface. To measure height in children over the age of 2, children were assessed standing with their heels, buttocks, shoulders and head making contact with a wall. The interviewer marked their height against the wall using a book or ruler and masking tape. All were measured barefoot and without hair adornments. Age- and sex-standardized body mass index (BMI) z-scores were calculated using standard techniques,[49] and a binary variable was created to indicate normal weight or overweight/obese using World Health Organization definitions.[50] Underweight children were excluded from the sample (<1% of children). While different cut-offs are generally used for 6 year olds,[51] we applied the same cut-offs to the entire sample population to facilitate comparisons across ages.

#### Externalizing and internalizing behaviors

Our main outcome variables measured child behavior. The Child Behavior Checklist (CBCL) measures externalizing and internalizing behaviors as reported by a parent of the child during in-home interviews.[52] There is strong evidence supporting the reliability and validity of the preschool- and school-age versions of the CBCL.[53–55] The CBCL has been shown to work well in a wide variety of countries, including Chile.[56,57] The preschool-age checklist was used for children 1.5 to 5 years of age. Externalizing subscales included aggressive behaviors and attention problems. Internalizing subscales included anxiety/depression, emotional reactivity, withdrawal and somatic symptoms. The school-age checklist was applied to children 6 years of age. This checklist included many of the same questions as the preschool-age checklist, but also had items related to socialization and following instructions, which are more relevant for children as they enter formal schooling situations. Externalizing subscales include aggressive behavior and disruptive conduct. Internalizing subscales include anxiety/depression, withdrawal and somatic symptoms. Because there is no international standard with which to compare, our sample served as its own reference. Age-adjusted z-scores were calculated within two-month age segments using the means and standard deviations from each age segment. The scores were centered on zero with a standard deviation of one. Higher scores indicated more externalizing and internalizing problems.

#### Covariates

The primary caregiver provided information on the child’s sex, indigenous status (self reported belonging to or descending from one of the nine legally recognized indigenous groups of Chile), birth weight (referring to the birth certificate when available), and the number of months the child was exclusively or partially breastfed. The primary caregiver was weighed and measured during the interview, and her BMI was calculated. She was also asked to report level of education completed (primary education, completed secondary education, some higher education but did not receive a degree, and completed higher education). Wealth is a measure of household financial and physical resources and is often used as a proxy for socioeconomic status.[58] In order to determine a family’s wealth, we conducted a principal components analysis of household assets (refrigerators, washing machines, cell phones, internet access, computers, DVD players, microwaves, hot water heaters, video cameras, and cable connections), a standard approach.[59] After rotating the factor loads using the varimax rotation, the first principal component was primarily defined by the presence of a computer, cellular phone and having an internet connection in the household. The first principal component was retained and split into quintiles for use in the models. Birth weight, months breastfed, primary caregiver’s BMI, and wealth were all included as continuous variables in the models. Birth weight and primary caregiver’s BMI were mean-centered.

#### Effect-measure modifiers

The child’s age (in years) and sex were explored as potential effect-measure modifiers. These characteristics were chosen because of their demonstrated role in modifying the association between weight and behavior among slightly older samples.[7] Child’s age was calculated from the birthdate recorded in the Civil Registry and the date of the psychological assessment.

### Statistical Model

We used multivariable linear regression to estimate the differences in CBCL scores comparing overweight or obese children to normal weight children, while controlling for the potentially confounding effects of demographic variables (child’s sex, indigenous status, birth weight, and months breastfed; primary caregiver’s BMI and education; and household wealth). Analyses were cluster-adjusted using linearized variance estimation to account for unobserved similarities among children from the same geographic area.[60] To examine the associations between weight and behavioral development across ages, we included interaction terms in our model, multiplying weight status by indicator variables for child’s age in years. We also assessed whether the timing of the association between weight status and behavioral problems varied by child sex using three-way interactions. To do so, we added interaction terms multiplying weight status by the indicators for age and sex as well as interaction terms for each unique pairing of these three variables to our main effects models. We used a p-value of 0.1 to determine significance for all interactions as studies are frequently underpowered to account for interaction.[61]

After seeing very little variation in the association between weight and behavior among the children ages 1 to 5 years old, we decided to conduct post-hoc tests of interactions using age as a binary variable (1–5 vs. 6 years). Rather than multiplying weight status by indicator variables for each year of age, these interactions terms included a binary variable indicating whether the child was 1–5 or 6 years of age. Post-hoc analyses of interactions also provided group-specific associations between weight status and behavior. We used a p-value of 0.05 to determine significance for within-group differences. We retained all potential confounders in our models based on their hypothesized associations with the exposure and outcome.

Because of the complexity of the sampling scheme, weights were included in the dataset to account for sampling probabilities and nonresponse. Applying the survey weights to the individual observations allows for extrapolation to the population of Chilean children born between 2006 and 2011.

We conducted a sensitivity analysis to assess how using the same weight cut-offs for the entire population of children versus using separate cut-offs for children aged 6 influenced the results. All analyses were conducted using Stata 12 (StataCorp. 2011. Stata Statistical Software: Release 12. College Station, TX: StataCorp LP).

## Results

Approximately 24% of the sample was overweight or obese, and the proportion was higher among older children (31%) (Table 1).

**Table 1:** Distribution of weight status among children in 2012.

	1.5–2 Years of Age		3–4 Years of Age		5–6 Years of Age	
	Frequency	Percent	Frequency	Percent	Frequency	Percent
Underweight	20	1%	76	1%	24	1%
Normal weight	1,394	75%	4,731	78%	3,050	67%
Overweight	326	18%	963	16%	1,019	23%
Obese	110	6%	318	5%	359	8%
N	1,850		6,088		4,452	

Sampling weights were used to generate the proportions in order to extrapolate to the population level. Age specific body mass index cut-offs were used to define weight categories according to World Health Organization definitions [50,51]. All children with weight data in 2012 included.

For the purposes of exploring associations, all following references to weight status refer to weight categories that were created using the same age- and sex-standardized BMI z-score cut-offs for the entire sample of children. Overweight or obese children were slightly younger, had higher birth weights, and had mothers with less education and higher BMIs compared with children in the normal weight range; however, the magnitudes of these differences were small (Table 2).

**Table 2:** Demographics by child weight status (N = 11207).

	Normal Weight	Overweight or Obese	P-Value
	(n = 8832)	(n = 2375)	
**Child Characteristics**			
Male–n (%)	4366 (50)	1215 (51)	0.158
Indigenous–n (%)	832 (10)	245 (10)	0.53
Age–n (%)			
1–2 years	1255 (30)	390 (34)	
3–4 years	4341 (41)	1200 (41)	0.01
5–6 years	3236 (30)	785 (26)	
Birth Weight (lbs)–Mean ± s.d	7.4 ± 1.1	7.6 ± 1.1	<0.001
Months Breastfed–Mean ± s.d	14.6 ± 11.7	14.0 ± 11.4	0.058
**Primary Caregiver Characteristics **			
Mother–n (%)	8762 (99)	2346 (99)	0.1157
Education–n (%)			
Primary or Less	1493 (15)	418 (16)	
Secondary School	5552 (62)	1531 (65)	0.0409
Higher Education: Incomplete	747 (8)	198 (8)	
Higher Education: Complete	1040 (14)	228 (11)	
BMI (kg/m^2^)–Mean ± s.d.	27.6 ± 5.0	29.2 ± 5.4	<0.001
**Household Characteristics **			
Wealth Factor^a^	0.0002 ± 1.0	0.003 ± 1.0	0.861

Sampling weights were used to generate the proportions in order to extrapolate to the population level. P-values were calculated using F tests and adjusted for clustering at the community level.

^a^ The wealth variable was constructed using principal components analysis and the first factor was retained.

We compared the children in the sample to children that were excluded from the sample because of missing data. Children in the sample were slightly younger on average (52.3 months compared to 53.9 months), were breastfed for longer (14.5 months compared to 12.4 months), and had mothers with slightly less education (63% completed secondary school compared to 58%) and lower BMIs on average (27.9 compared to 28.8) (Table 3).

**Table 3:** Demographic data comparing children in the sample to those with incomplete data.

	In Sample	Not in Sample	P-Value
	(n = 11207)	(n = 6002)	
**Child Characteristics**			
Weight Status			
Normal Weight	8832 (79)	851 (80)	
Overweight	1962 (18)	175 (16)	0.644
Obese	413 (4)	37 (3)	
Male–n (%)	5581 (50)	3117 (52)	0.004
Indigenous–n (%)	1077 (10)	389 (10)	0.186
Age–n (%)			
1–2 years	1645 (15)	344 (15)	
3–4 years	5541 (49)	921 (41)	<0.001
5–6 years	4021 (36)	960 (43)	
Birth Weight (lbs)–Mean ± s.d	7.5 ± 1.1	7.5 ± 1.1	0.017
Months Breastfed–Mean ± s.d	14.5 ± 11.6	12.4 ± 10.6	<0.001
**Primary Caregiver Characteristics**			
Mother–n (%)	11108 (99)	5915 (99)	0.001
Education–n (%)			
Primary or Less	1911 (17)	1017 (17)	
Secondary School	7083 (63)	3487 (58)	<0.001
Higher Education: Incomplete	945 (8)	609 (10)	
Higher Education: Complete	1268 (11)	855 (14)	
BMI (kg/m^2^)–Mean ± s.d.	27.9 ± 5.2	28.8 ± 5.7	0.003
**Household Characteristics**			
Wealth Factor^a^	0.001 ± 1.0	0.07 ± 1.1	0.014

Those “in sample” have complete covariate data. Those “not in sample” have incomplete covariate data or missing Child Behavior Checklist scores. The size of the population that was age-eligible to take the Child Behavior Checklist is approximate because exact age data were not available for children that did not take the test. P-values were calculated using F tests and adjusted for clustering at the community level.

^a^ The wealth variable was constructed using principal components analysis and the first factor was retained.

Comparing children of the same age, the differences in scores between normal weight and overweight/obese children among children ages 1 through 5 were uniformly small in adjusted and unadjusted analyses (<0.10 SD) and none of the weight-by-age interaction terms reached significance (data not shown). After grouping the 1 to 5 year olds to create a binary age variable (1–5 vs. 6 years), the differences in CBCL scores for the younger age group remained small, though after adjustment the difference in internalizing scores became statistically significant (Table 4). Overweight or obese 6 year olds had more total behavioral problems than normal weight 6 year olds (*β* = 0.16 SD, 95% CI = 0.013, 0.307, *P* = 0.033), and the difference in scores remained significant after adjustment for confounders (Table 4). The weight-by-age interaction term confirmed these findings and was significant for the total score (*β* = 0.17, 90% CI = 0.022, 0.315, *P* = 0.058). This finding can be interpreted to mean that the difference in behavioral scores by weight status among 6 year olds was significantly greater than the difference in behavioral scores by weight status among the 1 to 5 year olds.

**Table 4:** Adjusted and unadjusted differences in mean CBCL scores comparing overweight/obese and normal weight children (N = 11207).

*Unadjusted Model*			
	**Total**	**Externalizing**	**Internalizing**
Age 1–5			
Overweight	0.005	0.019	-0.052
95% CI	(-0.053, 0.063)	(-0.037, 0.075)	(-0.110, 0.006)
Age 6			
Overweight	0.160*	0.102	0.044
95% CI	(0.013, 0.307)	(-0.050, 0.254)	(-0.093, 0.181)
*Adjusted Model*^*a*^			
	**Total**	**Externalizing**	**Internalizing**
Age 1–5			
Overweight	-0.005	0.007	-0.062
95% CI	(-0.066, 0.055)	(-0.052, 0.065)	(-0.122, -0.001)
Age 6			
Overweight	0.163*	0.108	0.038
95% CI	(0.004, 0.322)	(-0.058, 0.274)	(-0.104, 0.181)

Sampling weights were used to generate the proportions in order to extrapolate to the population level. Both models adjusted for municipality-level clustering. Among 1–5 year olds n = 9,726. Among 6 year olds, n = 1,481.

^a^ Adjusted for child’s breastfeeding length, birth weight, sex, and indigenous status; household wealth; primary caregiver’s BMI and level of education.

* P-value<0.05

**Fig 1:**
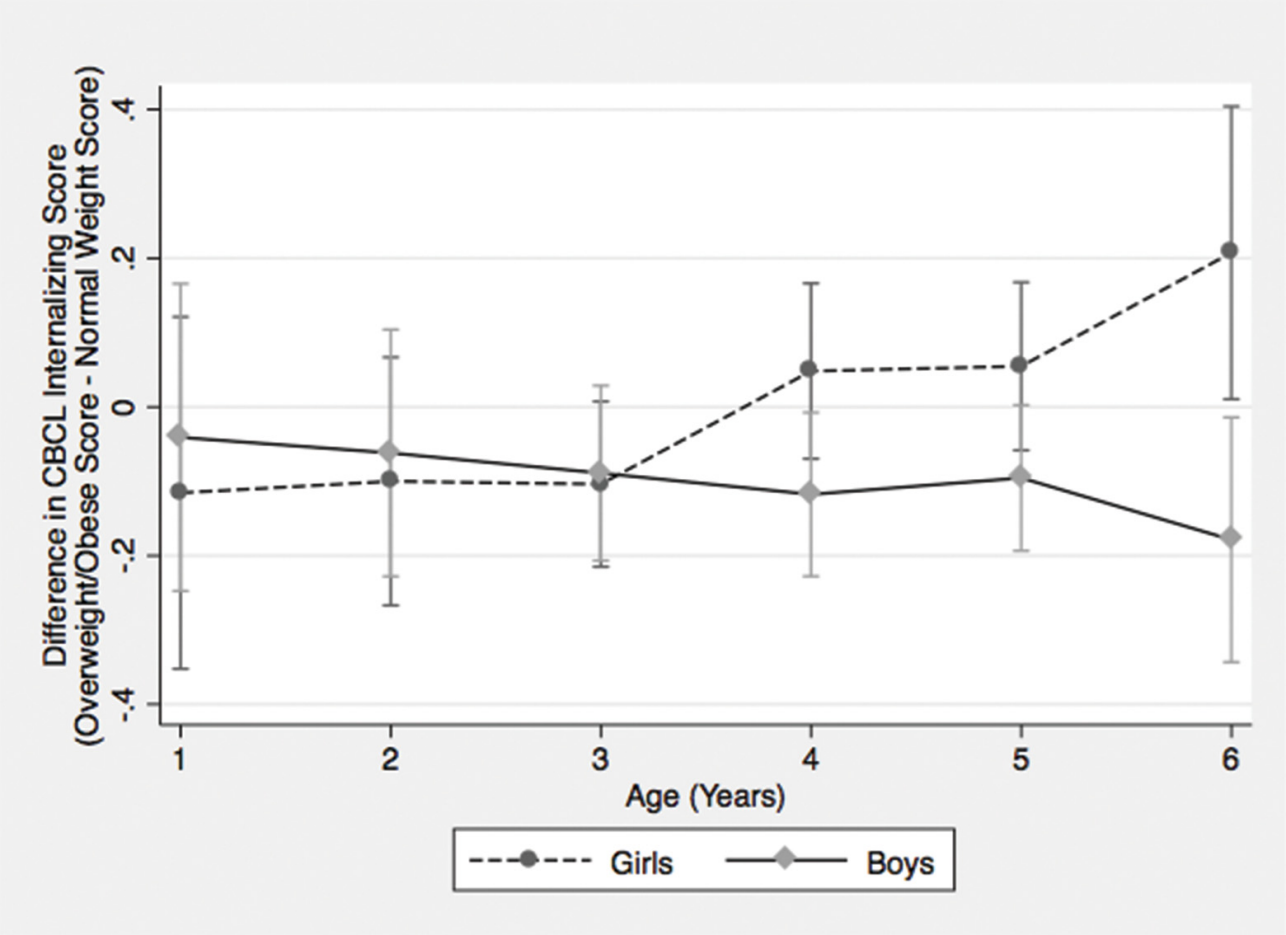
Difference in mean CBCL internalizing scores between overweight/obese and normal weight children by age and sex. Error bars are 90% CI. Age-adjusted z-scores were calculated using two-month age-specific means and standard deviations for the Child Behavior Checklist (CBCL) Internalizing score. The scores are centered on zero with a standard deviation of one.

In analyses of the association of weight within categories of sex, overweight or obesity was associated with more behavioral problems among 6 year old girls but not boys (Table 5). While no other within-group differences were statistically significant for the girls, the trend suggested overweight/obesity was associated with greater behavioral problems among girls than among boys. In fact, among boys ages 1 to 5 years, overweight/obesity was associated with a small reduction in internalizing behaviors (*β* = -0.09 SD, 95% CI = -0.163, -0.006). This sex difference was further supported by a significant weight-by-age-by-sex interaction on the internalizing scale, which showed the association between overweight/obesity and internalizing behaviors was statistically significantly greater for 6 year old girls than for boys of the same age (Fig 1).

**Table 5:** Adjusted differences in mean CBCL scores comparing overweight/obese and normal weight children by child’s age and sex (N = 11,207).

	Total		Externalizing		Internalizing	
	Girls	Boys	Girls	Boys	Girls	Boys
Age 1–5						
Overweight	0.015	-0.024	0.016	-0.003	-0.037	-0.085*
95% CI	(-0.070, 0.100)	(-0.106, 0.057)	(-0.068, 0.101)	(-0.085, 0.080)	(-0.119, 0.045)	(-0.163, -0.006)
Age 6						
Overweight	0.270*	0.028	0.151	0.055	0.207	-0.178
95% CI	(0.047, 0.493)	(-0.173, 0.230)	(-0.073, 0.374)	(-0.134, 0.243)	(-0.028, 0.442)	(-0.375, 0.018)

Sampling weights were used to generate the proportions in order to extrapolate to the population level. Adjusted for municipality-level clustering. Adjusted for child’s breastfeeding length, birth weight, and indigenous status; household wealth; and primary caregiver’s BMI and level of education. Among 1–5 year olds n = 9,726. Among 6 year olds, n = 1,481.

* P-value<0.05

We repeated these analyses using separate age- and sex-standardized BMI cut-offs for the 6 year olds,[51] which have lower thresholds for classifying children as overweight or obese than the standards for children aged 5 and under.[62] We found results for 6 year olds were attenuated, but when disaggregated by sex, the patterns from the main analysis remained. Differences in scores between normal weight and overweight/obese children among children aged 1 to 5 remained small and non-significant. Using the binary age variable (1–5 vs. 6 years), the pattern from the main analysis remained the same for the children in the younger age group, but differed for the 6 year olds. Whereas overweight or obese 6 year olds showed more total behavioral problems than their normal weight peers in the main analysis, the differences were not statistically significant when using separate age- and sex-standardized BMI cut-offs for the 6 year olds. Within categories of sex, overweight or obesity was associated with more externalizing in addition to total behavioral problems among 6 year old girls but not boys, and overweight/obesity continued to be associated with a reduction in internalizing behaviors among boys. Finally, using separate BMI cut-offs for the 6 years olds, the association between overweight/obesity and all three measures of behavior were statistically significantly greater for 6 year old girls than for boys of the same age.

## Discussion

Our results show that being overweight or obese at age 6 was associated with having more total behavioral problems, but that being overweight or obese at a younger age was not associated with having behavioral problems. These findings were driven by girls, and were not significant for boys. A few studies have found no association between weight and behavior among children under 4 years.[19,32] But some have found a significant association at this early age, such as a study showing an association between weight and behavioral development at 2 years old.[31] Another study among 4 and 5 year olds demonstrated small effect sizes in the relationship between weight and behavior.[63] In a study among kindergarteners in the US, being overweight was associated with an increased odds of teacher-reported externalizing and internalizing problems among girls; in that context, overweight and normal weight boys showed no statistically significant differences regarding emotional or behavioral problems.[30] A study among 5-year-old girls also showed that higher weight status was associated with lower body esteem. However this study sample did not include boys, nor children under the age of 5 years.[26] Another study found a small but marginally significant association between weight status and internalizing behaviors among kindergarteners. The magnitude of this association grew as the children aged through the third grade. However, unlike our findings, this study found child sex modified the association between weight and externalizing behaviors and not internalizing behaviors.[21] This evidence, combined with our findings, suggests that the strength of the association grows as children age, perhaps most notably among girls, and small differences at early ages may be hard to detect.

Our sensitivity analysis used different cut-offs to determine overweight and obesity among the 6 year olds. Because these cut-offs have a lower threshold for categorizing children as overweight or obese, the lack of an association between weight and behavior among the 6 year olds in this analysis could indicate that the association is only present among children with more extreme weights. Sex differences remained, however, with overweight/obesity being associated with more behavioral problems among 6 year old girls but not boys. The robustness of this finding suggests that the association between weight and behavior among 6 year olds is stronger for girls.

Our study adds to the literature by focusing on early childhood and a Latin American country. Additionally, we were able to explore the association by age and sex, providing a rich level of detail. This study also benefits from the inclusion of a validated measure of emotional and behavioral problems, the application of rigorous methodology in measuring weight and height, and the inclusion of a large, representative sample of children.

In spite of its strengths, our study is subject to some limitations. Our measures of emotional and behavioral development rely on parental reports, which could have led to measurement bias. Additionally, the subscales that comprise the externalizing and internalizing scales of the CBCL are slightly different for the 6 year olds than for the rest of the sample. If these subscales are capturing a different behavioral construct, we may be measuring a slightly different association among the 6 year olds. Given that the CBCL is designed to capture emotional and behavioral problems using age-relevant indicators, it is only natural that the assessment would change as children age in order to measure these latent constructs. Not all children had complete data, leading to the exclusion of a part of the original sample. Such underrepresentation could affect the generalizability of our results. After comparing those included in the final sample to those that were not included, we feel confident that the sample is representative of the broader population (see Table 3). Because the data are cross-sectional, we cannot determine whether weight status preceded behavioral problems or the reverse. Additionally, while effect modification by age suggests the association changes over time, longitudinal data would be better suited to address this question. The cross-sectional and observational nature of these data also precludes us from drawing any causal conclusions.

Developmental theory may offer insight into why we do not see an association between weight status and behavioral problems except among the 6 year olds. Evidence suggests that children are not able to imagine what others think and perceive until approximately age 6.[64] As a result, children younger than 6 may not be aware of more subtle messages about their weight status and the associated stereotypes. Even if they are aware of the messages, children may not use those external sources of information to describe themselves until they reach middle childhood.[65] In early childhood, children are still developing the ability to draw comparisons between their own and others’ abilities.[66]

Girls may be experiencing more pressure to conform to a body ideal at a younger age than boys,[67] which could explain why we see a more concerning association between overweight/obesity and behavioral problems among 6 year old girls. The social pressure for thinness is apparent in Chile. While body size is on the rise in Chile, body size ideals in Chile favor more slender shapes, especially for women.[68] According to a study of body size attitudes among middle-school-age students in Chile and other Latin American countries, more girls than boys wanted to be thinner even though the majority already perceived themselves as thin.[68] This preference for thinness seems to apply to younger children as well. In one study of mothers of primary-school children in Chile, mothers of overweight children showed more concern about their children’s weight than mothers of normal weight children.[69] In a study of mothers of children aged 18 to 36 months, the mothers of obese children reported dissatisfaction with their children’s weights, preferring that their children be thinner.[70] Girls may be subjected to more negative weight-related messages than boys, so that as children develop the ability to understand and internalize others’ judgments about them, the messages girls receive are more likely to cause them to internalize negative stereotypes.

If the association between weight and behavioral problems is largely due to poor impulse control, it may be that children do not gain sufficient autonomy over their food consumption until older ages, thereby masking the effect of poor impulse control on weight in early childhood.

This study contributes to the existing literature by identifying that weight status and emotional and behavioral development are correlated among child of an age to start elementary school, but not among younger children. This trend may mark the beginning of an association that grows as children age, playing an important role in children’s continued development, behavior and performance in school. There are clear strengths of the study, including the use of a large, nationally representative sample of Chilean children. However, the strength of the association was small, and the data were cross-sectional. Future research could shed more light on the associations between weight status and behavior by using longitudinal data to confirm how the association between weight status and behavioral problems changes as children age. Additionally, we would benefit from studies that directly measure the experience of weight-based stigma to explore whether it mediates the relationship between weight status and behavioral development or that measure child eating behaviors and parental feeding practices to explore their role as potential mediators in the behavior to weight status pathway.
